# Simplified Analytical Model for Predicting Neutral Cross-Section Position of Lenticular Deployable Composite Boom in Tensile Deformation

**DOI:** 10.3390/ma14247809

**Published:** 2021-12-16

**Authors:** Li-Wu Wang, Jiang-Bo Bai, Yan Shi

**Affiliations:** 1 School of Civil Engineering, Southeast University, Nanjing 210096, China; wangliwujinjin@126.com; 2 Beijing Institute of Space Mechanics & Electricity, Beijing 100094, China; 3 School of Transportation Science and Engineering, Beihang University, Beijing 100191, China; 4 Jingdezhen Research Institute of Beihang University, Jingdezhen 333000, China; 5 School of Automation Science and Electrical Engineering, Beihang University, Beijing 100191, China

**Keywords:** neutral cross-section, composite, lenticular, deployable boom

## Abstract

Foldable and deployable flexible composite thin-walled structures have the characteristics of light weight, excellent mechanical properties and large deformation ability, which means they have good application prospects in the aerospace field. In this paper, a simplified theoretical model for predicting the position of the neutral section of a lenticular deployable composite boom (DCB) in tensile deformation is proposed. The three-dimensional lenticular DCB is simplified as a two-dimensional spring system and a rigid rod, distributed in parallel along the length direction. The position of the neutral cross-section can be determined by solving the balance equations and geometric relations. In order to verify the validity of the theoretical model, a finite element model of the tensile deformation of a lenticular DCB was established. The theoretical prediction results were compared with the finite element calculation results, and the two results were in good agreement.

## 1. Introduction

The limitation of vehicle storage space and the demand for the construction of large space structures (such as solar wings, reflector antennas, solar sails, etc.) has promoted research and applications in relation to foldable (deployable) structures. A foldable structure can be stored in a relatively small-volume space before launch, and can be deployed into a large structure its working state in space [1,2,3,4]. Since thin-walled deployable composite structures have excellent stiffness, strength and ultralight mass characteristics, these structures are potential solutions for large aerospace engineering structures [5,6]. A deployable composite boom (DCB) is a classic flexible structure with excellent mechanical properties. DCBs exhibit large elastic deformation during the entire folding process, and strain energy can be stored while folding. When the DCB needs to be deployed, it can realize the deploying function with the help of an auxiliary mechanism and its own storage of elastic strain energy. The concept of a lenticular DCB was proposed by the DLR. This is a thin-walled beam structure, similar to a lenticular shape. Due to its high storage ratio and excellent mechanical properties, the lenticular DCB provides a new technical approach for large-scale deployable aerospace engineering structures [7,8]. In the design of this kind of structure, one needs to pay attention to the problems of its large deformation ability (in folding and deploying) and its fully deployed state. In terms of technical realization, it is necessary to perform verification tests of its materials, mechanics, and fabrication, as well as functional and extreme environmental verification in relation to its large deformation function and high deploying performance, the electromechanical integration of the folding/deploying mechanism with flexible characteristics, etc.

Sikinger et al. [9,10,11] studied the axial compression and bending properties of a lenticular DCB using the finite element method. They found that the main failure mode of the lenticular DCB was buckling instability, and the lenticular DCB showed excellent buckling resistance, especially in terms of the buckling instability caused by the bending moment. Sikinger et al. [12,13] also studied the temperature distribution of a lenticular DCB in a space environment, observing the influence of surface coating effects on the temperature field, thermal deformation, vibration, and other properties, using the finite element method and an experimental method. Block et al. [7] investigated the inflation-assisted deployment process of a 14-meter-long lenticular DCB under simulated zero gravity on a large A300 passenger plane, and verified the influence of different deployment control methods on the deployment process. Bai et al., from Beijing University of Aeronautics and Astronautics [1], were the first to successfully design and prepare lenticular DCBs that satisfied the function of large deformation in China. They carried out systematic investigations on relevant technical problems and completed the development process, from reduced-size verification parts to large-size verification parts for engineering prototypes. An analytical model of in-plane strain and interlaminar shear stress of a lenticular DCB was established, and the effectiveness of the analytical model was verified through flattening and coiling tests [14]. The finite element model for calculating the folding deformation of a lenticular DCB has been established and compared with analytical and experimental methods [15]. The technology for the preparation of a lenticular DCB has been studied, and a novel preparation method for a lenticular DCB has been obtained [1]. Experiments simulating the space thermal environment of lenticular DCB have been completed, and a heat transfer analysis model of a lenticular DCB in the thermal environment of space was established and compared with the experimental results [16]. The influence of high and low temperatures on the axial compressive buckling performance of lenticular DCBs was also experimentally studied, the corresponding finite element model was established, and the mechanical mechanism of buckling was analyzed. The calculated results are in good agreement with the test results [17]. In addition, the design technology, preparation process and experimental verification technology for ultra-long DCBs have also been explored. At present, the fabrication capacity exists for 60-m ultra-long flexible composite structures, and an engineering prototype has been developed. Jia et al. [18] numerically investigated the nonlinear buckling and post-buckling behavior of a lenticular DCB under pure bending. Parametric analysis and design space generation have been carried out, and several key factors have been determined. Fernandez et al. [19,20,21] characterized the folding properties, deployed stiffness, and fabrication methods of DCBs. The possible engineering application of DCBs for solar sails, gossamer sail systems, etc., have also been explored. Yang et al. [22,23,24] established a finite element model for analyzing the coiling deformation of DCBs with triangular sections, C sections, and M sections. The structural optimization design of DCBs based on a surrogate model has also been carried out.

In summary, many investigations have been conducted on lenticular DCBs, mainly focuses on two aspects—their large deformation abilities and fully deployed performance. According to the actual needs of engineering, there are still many problems to be further studied. There have been some relatively mature investigations on the large deformation of lenticular DCBs in terms of analytical models, finite element and test methods, but the analysis of the neutral section position of the tensile deformation of a lenticular DCB has not been reported, and the neutral cross-section position is very important for the design of the folding mechanism and the accurate control of folding and deploying. Therefore, it is necessary to carry out the relevant research.

## 2. Simplified Analytical Model

**Figure 1:**
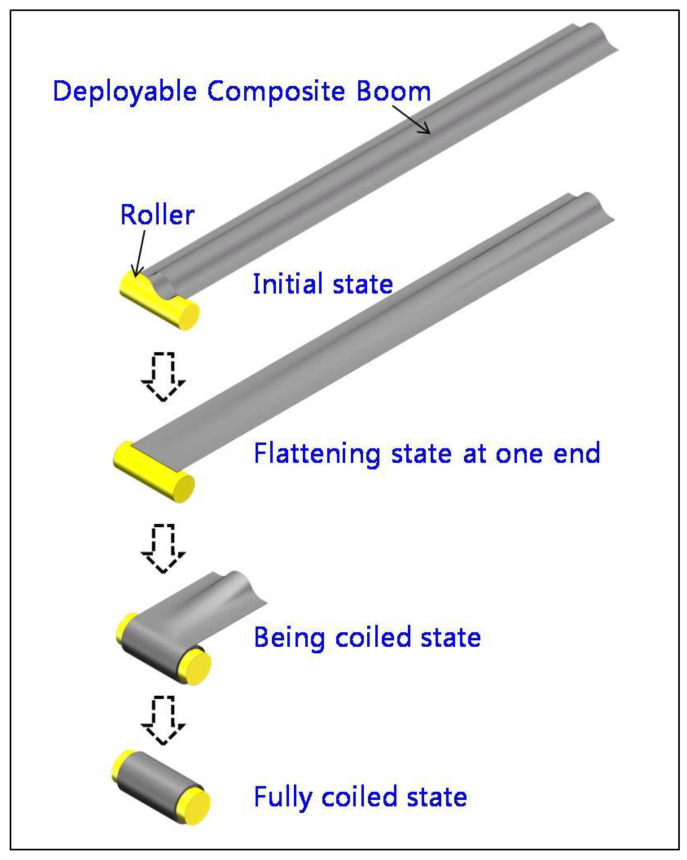
Folding deformation of a lenticular DCB.

**Figure 2:**
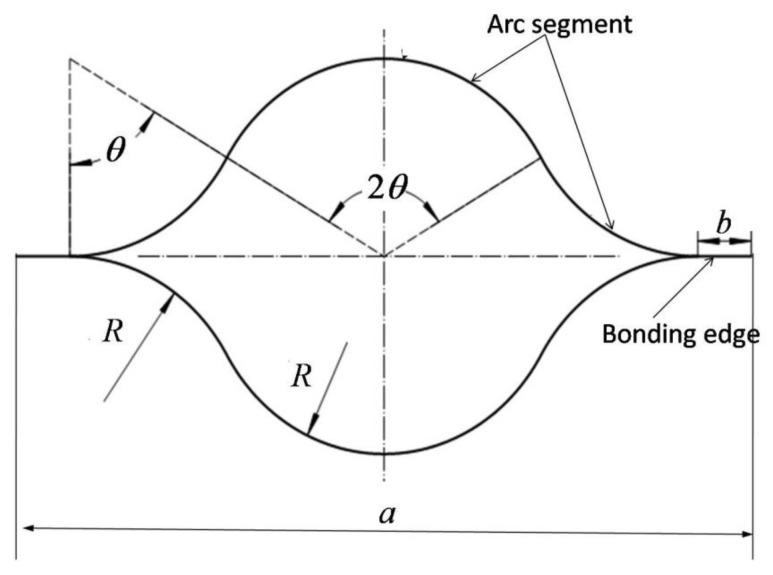
Geometric dimension of a lenticular DCB.

The lenticular DCB can realize the folding and deploying function through its own elastic strain, which is stored in the deformation process. In the first step, one end is stretched and deformed. In the second step, the folding or deploying process is completed through the roller. A schematic diagram of the entire deformation process is shown in Figure 1. The above process needs to be completed through the design of a suitable mechanism. The cross-sectional geometry of a lenticular DCB is shown in Figure 2, which is composed of an arc segment and bonding edge. In the first step of the tensile deformation process of the lenticular DCB, although the cross-section shape of the neutral cross-section remains unchanged, the cross-section shapes of the other positions along the length direction change. The cross-section from the neutral cross-section to the tensile end will be elongated in the width direction, and in particular, the cross-section from the neutral cross-section to the free end will be shortened. The position of the neutral cross-section is very important for the design of the mechanism. The mode of connection between the lenticular DCB and the coiling mechanism, the geometric dimensions of the coiling mechanism and the design of the root constraints in the fully deployed state all need to consider the position of the neutral cross-section. These detailed design steps for deployable mechanisms based on lenticular DCBs need to take into account the functions of folding and deploying and the DCB’s mechanical behavior in its fully deployed state.

**Figure 3:**
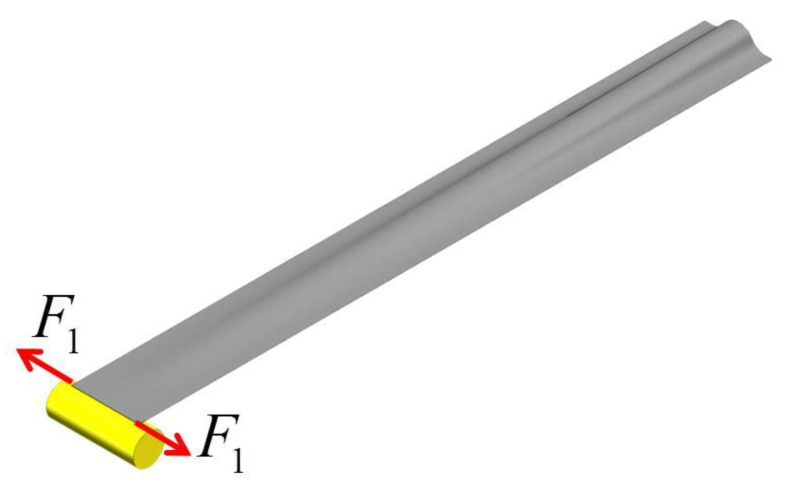
Tensile deformation process of a lenticular DCB.

**Figure 4:**
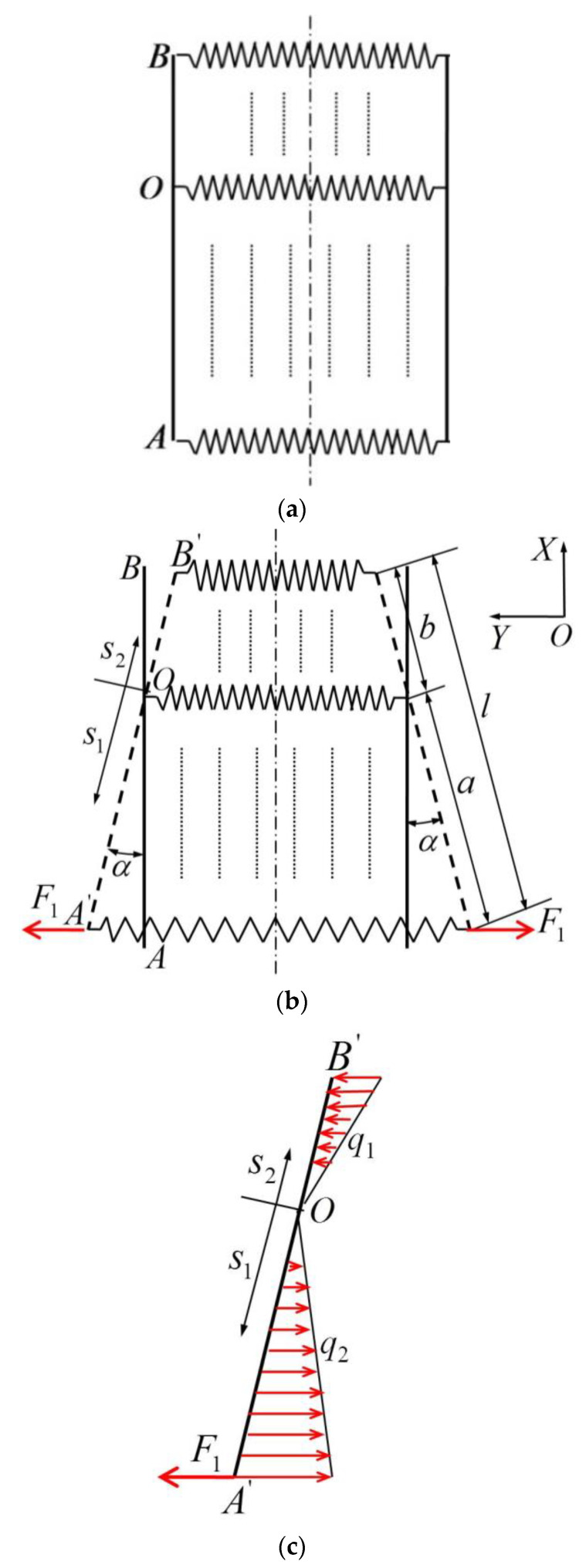
Idealized model of a lenticular DCB under tensile loading. (**a**)Iinitial state, (**b**) tensile state, (**c**) forces on the rod.

The lenticular DCB is subjected to transverse tensile load *F*_1_ (shown in Figure 3), and the loaded end is gradually flattened. The lenticular DCB, model under a transverse tensile load, can be simplified into a “spring system” (shown in Figure 4a) that is continuously distributed in parallel along the length direction. The stiffness per unit length of the arc segment (the “spring” part) is *k*, and the bonding edge AB is approximately a rigid rod. When a transverse tension is applied at one end, there is a rotation center O (shown in Figure 4a,b), where the “spring” remains in the initial stress-free state (i.e., the “spring” remains the original length), whereas the “springs” at both ends of the center are subjected to compression (OB part) and extension (OA part), corresponding to *q*_1_ and *q*_2_, respectively.

The equilibrium equations of the forces and moments shown in Figure 4c are expressed as

(1) $$\sum F_{y}=0$$

(2) $$\sum M_{o}=0$$

Specifically, this can be expressed as

(3) $$\int_{0}^{a}q_{2}ds_{1}-\int_{0}^{b}q_{1}ds_{2}=F_{1}$$

(4) $$\int_{0}^{a}q_{2}s_{1}\cos\alpha ds_{1}+\int_{0}^{b}q_{1}s_{2}\cos\alpha ds_{2}=F_{1}a\cos\alpha$$

where *F*_1_ is the transverse concentrated tensile load on the lenticular DCB. α is the corner of the bonding edge. $s_{1}$ and $s_{2}$ are length variables in two directions along the rotation center of the bonding edge. *a* is the total length of the “spring” part under tensile load. *b* is the total length of the “spring” part under compression load. *q*_1_ and *q*_2_ are respectively the loads corresponding to the compression (OB part) and tension (OA part) of the “spring”, which can be written as

(5) $$q_{1}=ks_{2}\sin\alpha$$

(6) $$q_{2}=ks_{1}\sin\alpha$$

where *k* is the stiffness per unit length of the “spring” part of the simplified model. For the calculation of this stiffness *k*, please refer to [25].

Substituting Equations (5) and (6) into Equations (3) and (4), we obtain

(7) $$\int_{0}^{a}ks_{1}\sin\alpha ds_{1}-\int_{0}^{b}ks_{2}\sin\alpha ds_{2}=F_{1}$$

(8) $$\int_{0}^{a}ks_{1}^{2}\sin\alpha \cos\alpha ds_{1}+\int_{0}^{b}ks_{2}^{2}\sin\alpha \cos\alpha ds_{2}=F_{1}a\cos\alpha$$

Integrating Equations (7) and (8) yields

(9) $$\frac{k_{1}a^{2}\sin\alpha }{2}-\frac{k_{1}b^{2}\sin\alpha }{2}=F_{1}$$

(10) $$\frac{k_{1}a^{3}\sin\alpha \cos\alpha }{3}+\frac{k_{1}b^{3}\sin\alpha \cos\alpha }{3}=F_{1}a\cos\alpha$$

Substituting Equation (9) into Equation (10), we obtain

(11) $$\frac{a^{2}-b^{2}}{a^{3}-b^{3}}=\frac{2}{3a}$$

where *a* and *b* are the lengths of the OA part and the OB part, respectively. The total length of the lenticular DCB is *l*, which can be expressed as

(12) a+b=l

Equations (11) and (12) are combined to form Equations about *a* and *b*, as follows:

(13) $$\left\{ \begin{array}{l} \frac{a^{2}-b^{2}}{a^{3}-b^{3}}=\frac{2}{3a}\\ a+b=l \end{array}\right.$$

The equations of Equation (13) can be solved as

(14) $$\left\{ \begin{array}{l} a=\frac{2l}{3}\\ b=\frac{l}{3} \end{array}\right.$$

Substituting Equation (14) into Equations (9) and (10) yields

(15) $$\left\{ \begin{array}{l} \sin\alpha =\frac{6F_{1}}{k_{1}l^{2}}\\ F_{1}=\frac{k_{1}l\Delta_{Ay}}{4} \end{array}\right.$$

where $\Delta_{Ay}$ is the lateral (Y-direction) displacement of end A.

In summary, according to Equations (14) and (15), the rotation center is at 2/3 of the length; that is, the position of the neutral cross-section of the lenticular DCB during tensile deformation is at 2/3 of the axial length direction.

## 3. Model Validation

### 3.1. Finite Element Model

**Figure 5:**
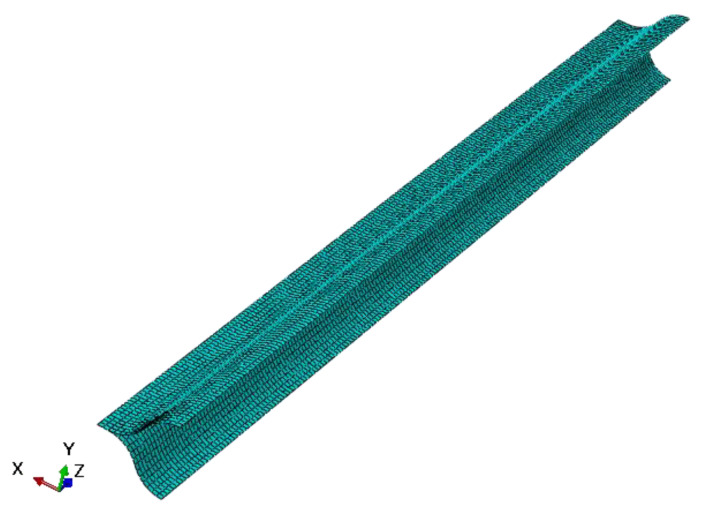
Finite element model of a lenticular DCB.

**Figure 6:**
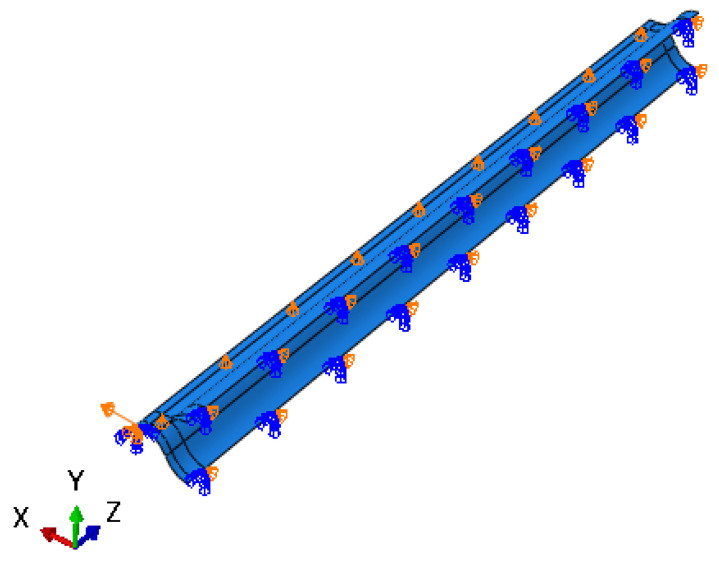
Boundary conditions of a lenticular DCB.

The position of the neutral cross-section predicted by the theoretical model is given in the previous section, and the theoretical model is verified via finite element (FE) calculation in this section. The FE model of the lenticular DCB was established using commercial software ABAQUS. In the example of the finite element calculation, the cross-section geometric parameters of the lenticular DCB are shown in Figure 2. The width b of the bonding edge is 10 mm, the radius of curvature *R* is 34 mm, the arc corresponding to radian *θ* is 60 degrees, the wall thickness is 0.4 mm, and the length *L* is 1000 mm. The ply scheme of the lenticular DCB is [45/−45/0/−45/45]. The mechanical properties of the composite ply are listed in Table 1, and these were obtained from experiments. Taking the above geometric parameters and material parameters as inputs, a three-dimensional finite element model (shown in Figure 5 and Figure 6) was established for the tensile deformation process of a lenticular DCB. Due to the symmetry of the ply and geometric configurations, only half of the model needs to be established. Symmetrical boundary conditions were applied at the symmetrical position. The tensile displacement load was applied at the end of one end, and the maximum tensile displacement was 12.5 mm, ensuring that one end of the lenticular DCB can be completely flattened. The element type of the finite element model of the lenticular DCB was S4R, and the total number of elements was 9750. The S4R element only takes into account the linear part of the nodal incremental displacement, thus significantly saving the calculation time [23]. In addition, this element type has also been successfully used in the large deformation analysis of thin-walled deployable structures [15,21,22,23,24]. The selected element size can ensure the accuracy and efficiency of the calculation results.

### 3.2. Calculation Results

**Figure 7:**
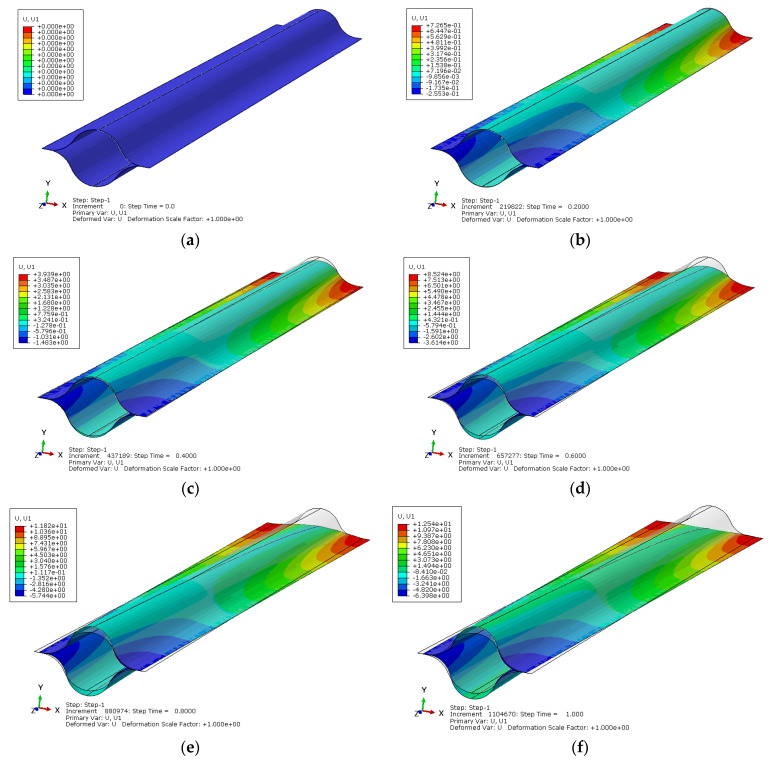
Simulated X-direction deformation of a lenticular DCB. (**a**) Initial state, (**b**) 20% of the maximum tensile deformation, (**c**) 40% of the maximum tensile deformation, (**d**) 60% of the maximum tensile deformation, (**e**) 80% of the maximum tensile deformation, (**f**) the maximum tensile deformation.

Using the established finite element model of the lenticular DCB during tensile deformation, the cross-section change along the length direction of the flattening deformation process can be calculated. The change can be quantitatively described by transverse displacement (i.e., displacement in the X direction). When the displacement in the X direction is greater than 0, it indicates that the cross-section at the corresponding position is stretched and widened. When the displacement in the X direction is less than 0, it indicates that the cross-section at the corresponding position is compressed and narrowed. Figure 7 shows the X-direction displacement nephogram of the lenticular DCB during flattening deformation. The gray contour line in the figure is the original configuration state before deformation. It can be seen in Figure 7 that the area near the applied tensile load is obviously stretched and widened, and the height direction becomes flat; the area near the free end is obviously compressed and narrowed, and the size in the height direction becomes larger. Moreover, there is a transition cross-section position, which is not stretched or compressed, namely, the cross-section shape does not change. We define this cross-section as the neutral cross-section. This is consistent with the phenomena predicted by the previous theoretical analysis.

**Figure 8:**
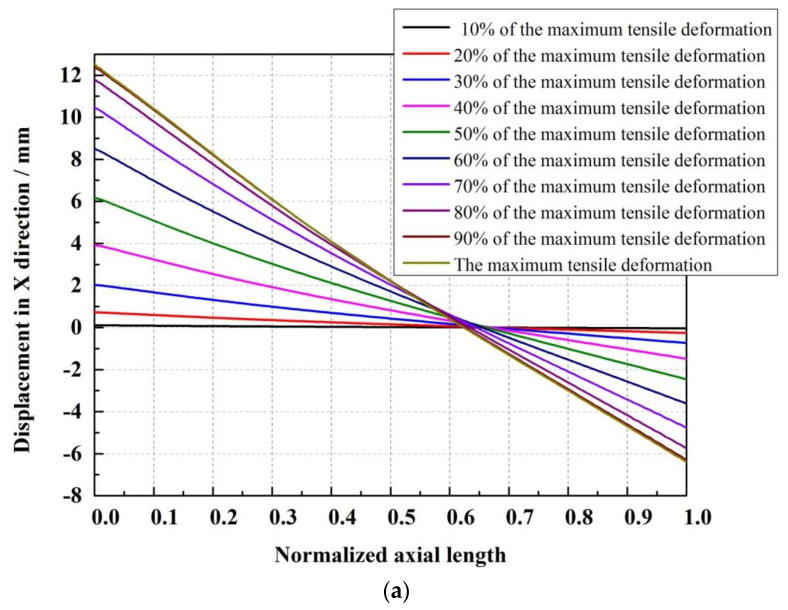

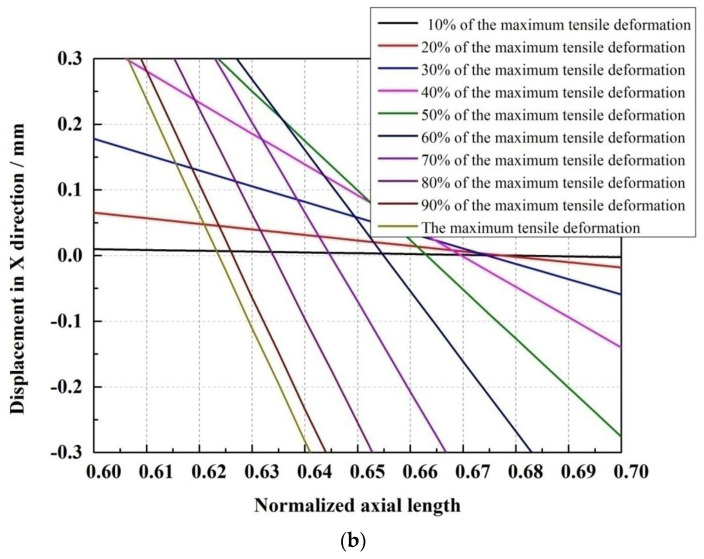
X-direction deformation of the bonding edge of a lenticular DCB. (**a**) Full range, (**b**) local range.

**Figure 9:**
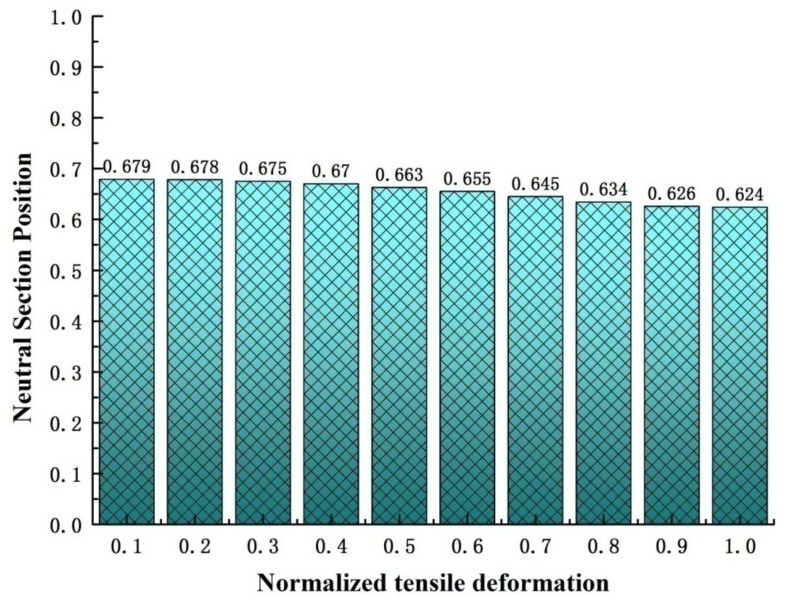
Neutral section position under different tensile deformation levels in a lenticular DCB.

In order to obtain the neutral cross-section position of the lenticular DCB during tensile deformation, the transverse displacement of each position corresponding to the length direction (z direction) of the bonding edge is the output, and the obtained curve is shown in Figure 8. Figure 8 shows the relationship between the normalized axial length (i.e., the z direction) of the bonding edge and the displacement in the X direction under different maximum tensile deformation conditions during the tensile deformation of the lenticular DCB. As can be seen from Figure 8a, under different tensile deformation conditions, there is a position of the 0 point in the X direction displacement, which is between 0.6 and 0.7 in the length direction. The corresponding position here is the position of the neutral cross-section. From Figure 8b, with the increase in tensile deformation, the neutral cross-section position point floats within a certain range (i.e., 0.624–0.679). The normalized axial length of the neutral cross-section position point decreases with the increase in tensile deformation, but the floating range is small, as shown in Figure 9. This study mainly considers flattening caused by tension. As long as one end remains in a completely flattening state, whether caused by tension or compression, the positions of the neutral cross-section are about at 2/3 of the length. However, in the case of compression, the deviation may be larger due to the local deformation of the arc segment contacting the compressive load and due to the influence of boundary conditions.

## 4. Discussion

The neutral cross-section position predicted by the theoretical model is at the position 2/3 of the normalized axial length. Therefore, according to the above quantitative analysis, the relative error range between the theoretical predictions and the finite element calculations were between 6.45% and 1.80%. The theoretical predictions were in good agreement with the finite element calculations. With the increase in the tensile deformation, the prediction accuracy of the theoretical model decreased. This is because in order to facilitate the calculation and simplified analysis, a simplified model—“spring system” was proposed in this paper. The one-dimensional spring response was used to approximate the elastic response of the circular arc segment, and the bonding edge was simplified into a rigid rod. When the tensile deformation increases, the elastic deformation of the bonding edge can also affect the position of the neutral section. With an increase in the elastic deformation of the bonding edge, the position of the neutral section will move forward, which is reflected in the finite element calculation results. If the elastic deformation of the bonding edge can be considered in the theoretical model, the prediction accuracy can be improved, but the complexity of the model will be significantly increased. More detailed large deformation calculations can be analyzed with the help of finite models, referring to the methods described in the relevant literature [15,22].

## 5. Conclusions

(1) A simplified theoretical model for predicting the position of the neutral cross-section of a lenticular DCB during tensile deformation was established. The position point of the neutral cross-section predicted by this theory was at the position 2/3 of the normalized axial length, and the shape of the neutral cross-section remained unchanged during tensile deformation. The width direction of the area near the applied tensile load was obviously stretched and widened, and the height direction became flat, whereas the width direction of the area near the free end was obviously compressed and narrowed, and the size in the height direction became larger.
(2) The tensile deformation of the lenticular DCB was calculated using the finite element method. It was found that under different tensile deformation conditions, there was a 0-point displacement in the X direction, which was between 0.624 and 0.679 in the normalized length direction. The normalized axial length of the neutral cross-section point decreases with an increase in tensile deformation, but the floating range is small.
(3) The relative error range between the neutral cross-section position predicted by the simplified analytical model and the finite element calculation results was between 6.45% and 1.80%, which shows good agreement, verifying the effectiveness of the theoretical model. However, the simplified theoretical model has not been verified by experiments.
(4) The finite element calculation showed that the elastic strain energy stored in the lenticular DCB increases with the increase in tensile deformation, and this is a nonlinear response relationship.

## Figures and Tables

**Table 1:** Mechanical properties of composite ply.

Property	Value
*E*_1_/GPa	80.08
*E*_2_/GPa	6.67
*v*_12_	0.344
*G*_12_/GPa	2.93
*G*_13_/GPa	2.93
*G*_23_/GPa	2.5

## Data Availability

The data presented in this study are available on request from the corresponding author.
